# Quantitative analysis of the root posture of *Arabidopsis thaliana* mutants with wavy roots

**DOI:** 10.1017/qpb.2024.12

**Published:** 2024-11-25

**Authors:** Hiroki Yagi, Ikuko Hara-Nishimura, Haruko Ueda

**Affiliations:** 1 Graduate School of Natural Science, Konan University, Kobe 658-8501, Japan; 2 Faculty of Science and Engineering, Konan University, Kobe 658-8501, Japan

**Keywords:** ATP-BINDING CASSETTE SUBFAMILY B19, curvature index, myosin XI, root posture, straightness index

## Abstract

Plant postures are affected by environmental stimuli. When the gravitational direction changes, the *Arabidopsis thaliana* mutants *myosin xif xik* (*xif xik*) and *atp-binding cassette b19* (*abcb19*) exhibit aberrantly enhanced organ bending. Whether their phenotypes are due to the same mechanism is unknown. We characterized the primary root postures of these mutants. Their roots exhibited enhanced gravitropic bending with the same root-tip angles. The wavy roots of vertically grown plants were quantitatively evaluated using four indices. The straightness index (root base-to-tip length to total root-length ratio) was similar for *xif xik* and *abcb19*, and it slightly decreased for *xif xik abcb19.* The curvature index was similar for *abcb19* and *xif xik abcb19*, but it decreased for *xif xik*, suggesting the ABCB19 deficiency caused the roots to curve more sharply. Combination of these indices for quantitative analyses of root postures may distinguish between similar wavy-root phenotypes and clarify genetic relationships.

## Graphical abstract

**Figure:**
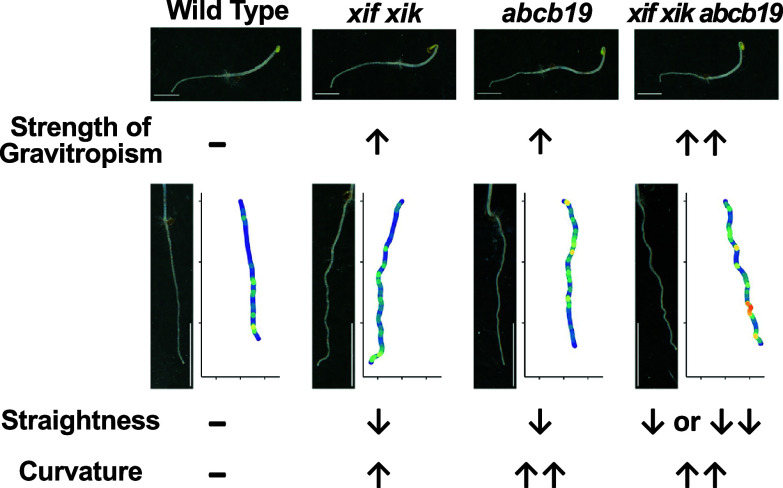

## 1. Introduction

Plant posture is influenced by various environmental stimuli, including gravity, light and touch (Darwin, 1880; Gilroy, 2008; Harmer & Brooks, 2018). Tropism refers to the directional movement of plant organs in response to stimuli. After plants perceive a change in the direction of gravity, polar auxin transport is altered (Žádníková et al., 2015; Nakamura et al., 2019). The differential distribution of auxin induces differential cell growth, leading to organ bending (Esmon et al., 2005a, 2005b). The primary inflorescence stems and the primary roots grow against and towards the direction of gravity, respectively. In *Arabidopsis thaliana*, mutations to auxin transporter genes result in aberrant responses to gravity (Müller et al., 1998; Marchant et al., 1999; Friml et al., 2002). For example, in *abcb19* mutants, the deficient expression of *ATP-BINDING CASSETTE SUBFAMILY B19* (*ABCB19*)/*MULTIDRUG RESISTANCE PROTEIN1* (*MDR1*)/*P-GLYCOPROTEIN 19* (*PGP19*), which encodes an auxin efflux transporter, leads to enhanced gravitropism (Noh et al., 2001, 2003; Geisler et al., 2005; Rojas-Pierce et al., 2007; Bailly et al., 2008), enhanced phototropism (Noh et al., 2003) and wavy roots and hypocotyls (Noh et al., 2003; Lewis et al., 2007; Nagashima et al., 2008; Zhao et al., 2013).

Previous studies on gravitropism revealed that tropisms are likely mediated by organ bending and organ straightening (Stanković et al., 1998; Bastien et al., 2013; Okamoto et al., 2015). The straightening process (i.e., autotropism or autostraightening) maintains a straight posture, probably through the perception of the degree of bending (Stanković et al., 1998; Haga & Iino, 2006; Okamoto et al., 2015). Although mathematical modelling studies have demonstrated the importance of the organ straightening process for controlling posture (Bastien et al., 2013, 2014; Moulton et al., 2020), the underlying molecular mechanism remains largely unknown. Intriguingly, organ straightening occurs without a reversal of the asymmetrical distribution of auxin during tropic responses (Haga & Iino, 2006). The only known regulator of organ straightening is the actin–myosin XI cytoskeleton. The *A. thaliana* mutants *myosin xif xik* (*xif xik*) and *actin8/frizzy1* are defective in the organ straightening process required for restoring posture (Okamoto et al., 2015). Various organs, such as roots, hypocotyls and inflorescence stems, of the *xif xik* mutants exhibit enhanced bending in response to gravity and light (Okamoto et al., 2015). Therefore, the *xif xik* and *actin8/frizzy1* plants have a kinked gross morphology (Okamoto et al., 2015; Ueda et al., 2015). By combining mathematical modelling and quantitative analyses of the shoot-bending behaviour of wild-type and *xif xik* plants, we recently demonstrated that the distribution of stress is modulated by the straightening process, resulting in a mechanically favourable shape (Tsugawa et al., 2023).

Quantitative analysis of root posture is an effective and easy means to determine the degree of organ straightening. Grabov et al. (2005) proposed the following three root posture indices: vertical growth index (*VGI*), horizontal growth index (*HGI*) and straightness (hereafter referred to as straightness index (*SI*)) (Grabov et al., 2005). Previous research indicated that *VGI* can distinguish the root posture of *tiny root hair1*, a gravitropism-deficient mutant, from that of the wild-type, but root length cannot (Vicente-Agullo et al., 2004; Grabov et al., 2005). Both *HGI* and *SI* were used for a quantitative trait locus analysis of the root morphology of *A. thaliana* accessions Landsberg *erecta* and Cape Verde Islands (Vaughn & Masson, 2011). In addition, these two indices were useful for differentiating between the root postures of the *A. thaliana* accessions Columbia-0 (Col-0) and Wassilewskija (Schultz et al., 2017). It is also reported that *SI* could be affected by the growth conditions including plant age (Grabov et al., 2005) and the presence of sucrose in the culture medium (García-González et al., 2022).

Exploration of the relationship between mutants with similar postures, such as aberrantly wavy roots, can be challenging. In this study, we aimed to establish simple phenotyping methods to evaluate plant posture using the readily manipulable roots of *A. thaliana*. First, to understand the genetic relationship between *MYOSIN XI* genes and *ABCB19* in posture control in response to gravity, we compared the gravitropic responses of roots of *xif xik* and *abcb19*, which have not been directly compared in previous studies. Second, we quantitatively compared the wavy posture of vertically grown roots of these mutants by calculating the curvature index (*CI*) in addition to the *VGI*, *HGI* and *SI*. The results indicated that a combination of these indices may enable the classification of similar wavy-root phenotypes and clarification of genetic relationships.

## 2. Materials and methods

### 2.1. Plant materials and growth conditions

*Arabidopsis thaliana* Col-0, *myosin xif-1 xik-2* (hereafter referred to as *xif xik*; Okamoto et al., 2015), two *abcb19* mutants (*abcb19-101*/*atmdr1-101* and *abcb19-102*/*atmdr1-102*; Lin & Wang, 2005) and the *xif xik abcb19-101* and *xif xik abcb19-102* triple mutants were used in this study. The *xif xik abcb19-101* and *xif xik abcb19-102* mutants were generated via genetic crosses. Seeds were surface-sterilized in 70% (v/v) ethanol and then aseptically sown on plates containing Murashige and Skoog medium (Nihon Pharmaceutical Co., Ltd., Tokyo, Japan) supplemented with 0.5% (w/v) gellan gum (FUJIFILM Wako Pure Chemical Corporation, Osaka, Japan), 0.5% MES-KOH (pH 5.7), 0.01% *myo*-inositol and with or without 1% (w/v) sucrose. The seeds were incubated at 4°C in darkness for a day to break dormancy, and then at 22°C under light condition for a day. Plants were grown almost vertically (pitches were 80–90°) along the surface of the medium at 22°C for 3 or 5 days in darkness to mitigate the effect of phototropism on root posture. Subsequently, the plants were used for a gravitropism assay or quantitative analysis of root posture.

### 2.2. Root gravitropism assay

**Figure 1.**
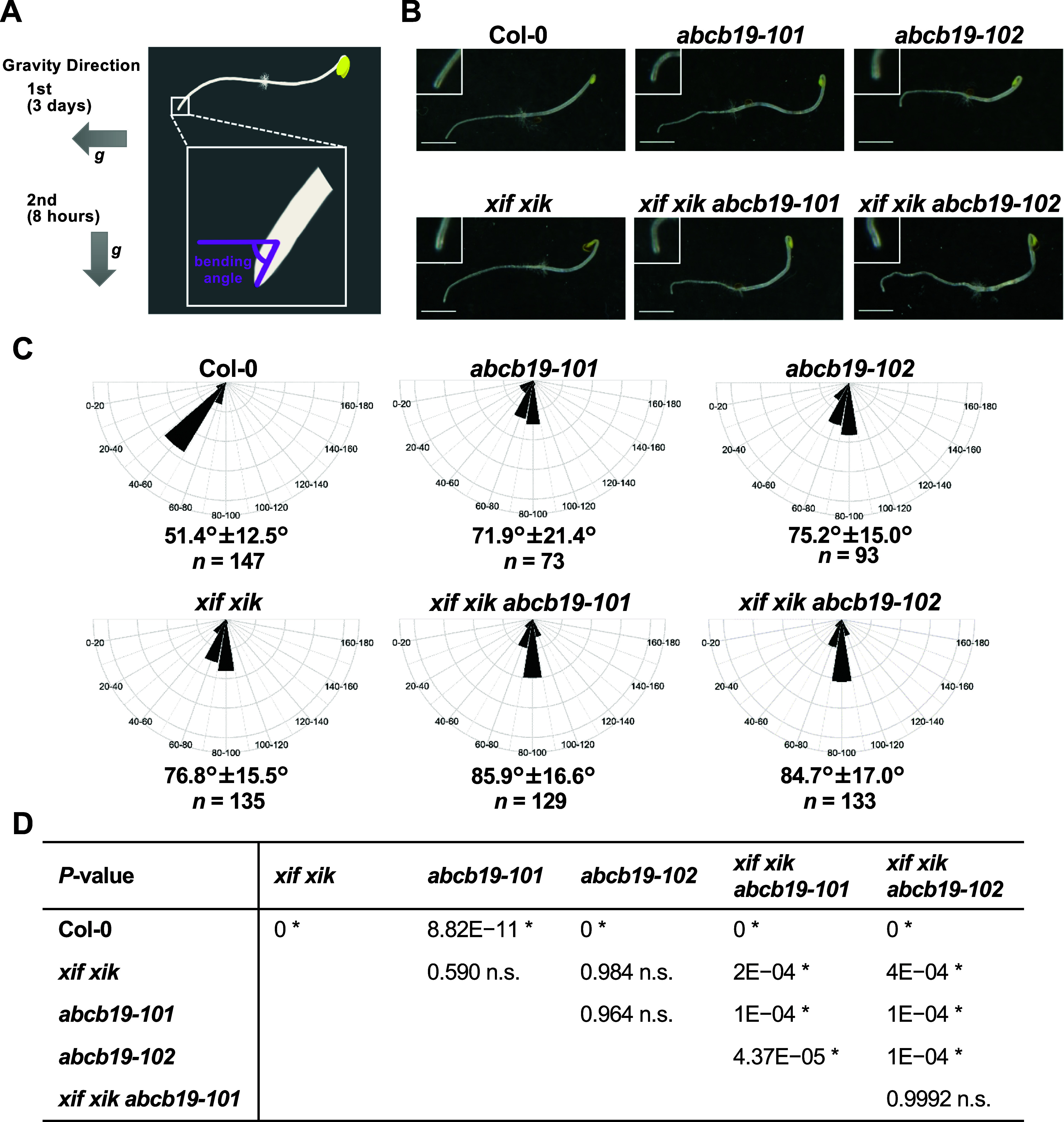
Measurement of gravitropic bending angles of roots. Three-day-old wild-type (Col-0), *myosin xif xik* (*xif xik*), *abcb19-101*, *abcb19-102*, *xif xik abcb19-101* and *xif xik abcb19-102* etiolated seedlings grown on agar medium in plates were reoriented by 90° in darkness. After gravistimulation for 8 hours, the plates were photographed. (a) Schematic diagram of an etiolated seedling after gravistimulation for 8 hours and the root-tip angle with a horizontal plane (purple circular arch). (b) Representative images of the wild-type (Col-0) and mutant plants. Magnified images of the root tips are shown in the inset images. Scale bars indicate 2.5 mm. (c) Circular histograms of the root-tip angles that were grouped into 20° classes. The mean ± standard deviation and sample number (*n*) are provided. (d) *P*-values for the differences in the root-tip angles between each pair of genotypes that were estimated by the Steel–Dwass test as a post hoc test following the Kruskal–Wallis ANOVA (*P*_ANOVA_ < 2.2E−16). *, *P* < 0.05; n.s., not significant.

Three-day-old etiolated seedlings grown on medium supplemented with sucrose in plates were reoriented by 90° in darkness. After gravistimulation for 8 hours, the plates were scanned with exposure to light using a scanner (GT-X830, EPSON, Nagano, Japan). The angle between the horizontal plane and the root tip was measured using ImageJ/Fiji v1.53f51/Java 1.8.0 software (Schindelin et al., 2012; National Institute of Health). Figure 1a shows a schematic illustration of measurement of the bending angle. The data were statistically processed using R v4.1.2 (R Core Team, 2021) and circular histograms were plotted using the “ggplot2” R package (Wickam, 2016). The sample sizes (*n*) were as follows: 147 (Col-0), 135 (*xif xik*), 73 (*abcb19-101*), 93 (*abcb19-102*), 129 (*xif xik abcb19-101*) and 133 (*xif xik abcb19-102*). The Kruskal–Wallis nonparametric one-way analysis of variance (ANOVA) was performed to analyse the differences among mean ranks as implemented in default R. The Steel–Dwass test was performed for post hoc nonparametric multiple pairwise comparisons with “pSDCFlig” function (method = “Asymptotic”) as implemented in the “NSM3” R package (Schneider et al., 2022).

### 2.3. Quantitative analysis of root posture

**Figure 2.**
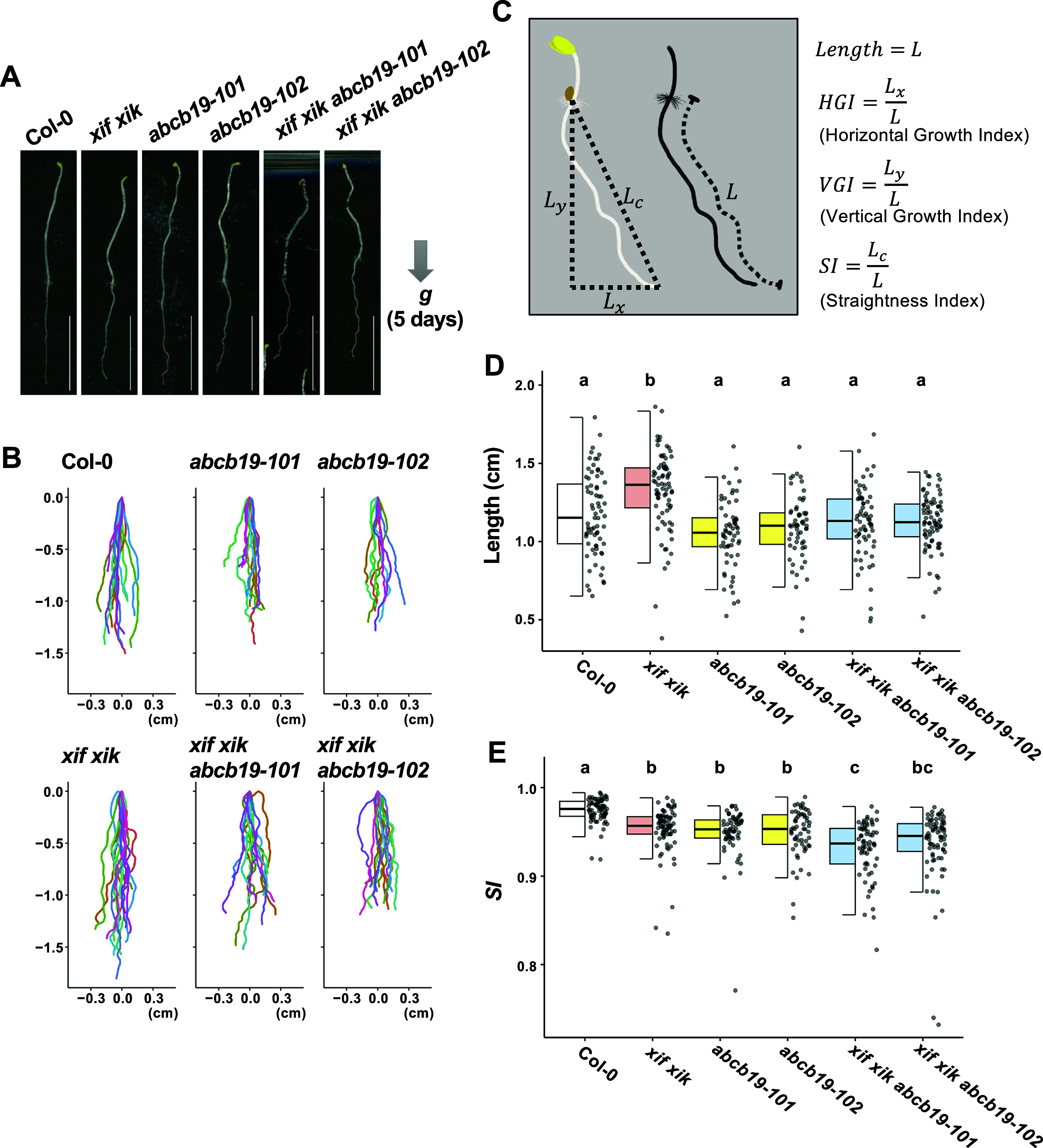
Evaluation of root postures in vertically grown seedlings by the straightness index (*SI*). Wild-type (Col-0), *myosin xif xik* (*xif xik*), *abcb19-101*, *abcb19-102*, *xif xik abcb19-101* and *xif xik abcb19-102* seedlings were grown vertically along the surface of the medium in darkness for 5 days. (a) Representative images of 5-day-old seedlings. Scale bars indicate 1 cm. (b) Posture plots of 15 individual roots for each genotype. The coordinate (0,0) indicates the basal point (the root–hypocotyl junction) of each root. (c) Schematic diagram of the root posture-related horizontal growth index (*HGI*), vertical growth index (*VGI*) and straightness index (*SI*). (d, e) Quantitative comparisons of root length (d) and *SI* (e) presented as box and dot plots. The bars indicate the sample ranges, each box indicates the first and third quantiles and the bold horizontal line is the median. The number of samples (*n*) is as follows: 71 (Col-0), 77 (*xif xik*), 62 (*abcb19-101*), 59 (*abcb19-102*), 66 (*xif xik abcb19-101*) and 78 (*xif xik abcb19-102*). Different letters (a, b and c) indicate a significant difference (*P* < 0.05; Steel–Dwass test following the Kruskal–Wallis ANOVA (*P*_ANOVA_ = 1.22E−11 in root length, and *P*_ANOVA_ < 2.2E−16 in *SI*)).

Plants were grown almost vertically on medium supplemented with or without sucrose in darkness for 3 or 5 days. The etiolated seedlings on the plates were scanned with exposure to light using a scanner. The “segmented line” regions of interest (ROIs) along the roots (from the root–hypocotyl junction to the root tip) were determined manually using ImageJ/Fiji v1.53f51/Java 1.8.0 software and transformed into “smoothed line” ROIs using the Fiji function “Fit Spline.” In total, 100–450 dots were marked along the root and then the coordinate data were exported to R v4.1.2. The sequential coordinates were fitted via spline interpolation to comprise 1,000 dots cm^−1^ using the “smoothr” R package (Strimas-Mackey, 2023). Supplementary Figure S1 summarizes the procedure to obtain the root coordinates. The primary root length (*L*), *VGI*, *HGI* and *SI* were calculated as described by Grabov et al. (2005) (Graphical definition of these indices is depicted in Figure 2c). In brief, *VGI* and *HGI* are the vertical and horizontal distances between the starting and ending points of the root (*L_y_* and *L_x_*) per *L*, respectively. *SI* is the distance between the starting and ending points (*L_c_*) per *L*. The *CI* was calculated as the ratio of the angle and distance between three sequential points. In other words, *CI* reflects the rate of change in the tangential angle of the curve. The *CI* of a straight line is 0, and the value increases as the sharpness of the curve increases. Next, a simple moving average of 0.005 cm before and after (0.01 cm in total) was used, considering the human error in the ROI setting in ImageJ/Fiji. The first and last 20 dots were eliminated from the curvature calculation. When calculating the mean *CI* for each root, the 1%-trimmed mean (the 0.5% lowest and highest values each were removed) was used. Root postures with or without *CI*-indicating colour were plotted on a two-dimensional plane using the “ggplot2” R package. All box and dot plots were generated using the “ggplot2” and “gghalves” R packages (Tiedemann, 2022). The sample sizes (*n*) for investigation of the effects of incubation time and sucrose on the root posture of Col-0 were as follows: 33 (3-day-old without sucrose; 3d−Suc), 29 (3-day-old with sucrose; 3d+Suc), 39 (5-day-old without sucrose; 5d−Suc) and 35 (5-day-old with sucrose; 5d+Suc). The Brunner–Munzel nonparametric test implemented in the “brunnermunzel” R package (Ara, 2022) with the Bonferroni correction was performed for comparisons of 3d−Suc versus 3d+Suc, 3d−Suc versus 5d−Suc, 3d+Suc versus 5d+Suc and 5d−Suc versus 5d+Suc. The data included outliers; however, the Brunner–Munzel test is robust to outliers and independent of homoscedasticity. To compare the root posture between genotypes, plants were grown on medium supplemented with sucrose for 5 days. The sample sizes (*n*) were as follows: 71 (Col-0), 77 (*xif xik*), 62 (*abcb19-101*), 59 (*abcb19-102*), 66 (*xif xik abcb19-101*) and 78 (*xif xik abcb19-102*). Statistical analysis of the data was performed using the Kruskal–Wallis nonparametric one-way ANOVA and the Steel–Dwass test for post hoc nonparametric multiple pairwise comparisons. It should be noted that the ANOVA *P*-values (*P*_ANOVA_) throughout the study, including the root gravitropism assay, were below the Bonferroni threshold of 0.05 (0.05/9 = 0.0056), except for *HGI* (Supplementary Figure S4a).

## 3. Results

### 3.1. Roots of xif xik and abcb19 mutants exhibited similar enhancement of gravitropic bending

We compared the root gravitropic response of the *xif xik* mutant, two *abcb19* mutants (*abcb19-101* and *abcb19-102*) and the *xif xik abcb19-101* and *xif xik abcb19-102* triple mutants. The bending of the primary roots and hypocotyls was greater for all mutants than for the wild-type (Col-0) control (Figure 1b). In this study, we focused on root posture because hypocotyls often did not elongate along the surface of the medium and were difficult to quantify. The Col-0 roots had the smallest bending angle (51.4 ± 12.5°; mean ± standard deviation) among the analysed genotypes (Figure 1c, d). The *xif xik*, *abcb19-101* and *abcb19-102* mutant roots had similar bending angles (76.8 ± 15.5, 71.9 ± 21.4 and 75.2 ± 15.0°, respectively; Figure 1c, d). Thus, following gravistimulation for 8 hours, the *xif xik* and *abcb19* roots were indistinguishable. The bending angles of the triple mutants *xif xik abcb19-101* and *xif xik abcb19-102* (85.9 ±16.6 and 84.7 ± 17.0°, respectively; Figure 1c, d) were significantly larger than those of *xif xik* and *abcb19*.

### 3.2. Roots of xif xik and abcb19 mutants had a similarly low SI

We noticed that, when seedlings were grown vertically on agar medium supplemented with sucrose in darkness for 5 days, the *xif xik* mutant had similar phenotypes to the *abcb19* mutants, which was in contrast to the straight roots and hypocotyls of the Col-0 plants (Figure 2a). Furthermore, the wavy phenotype was enhanced in the *xif xik abcb19-101* and *xif xik abcb19-102* mutants (Figure 2a). Plots of the coordinates for 15 representative root postures and for all root postures are presented in Figure 2b and Supplementary Figure S2, respectively. We first calculated three indices (*HGI*, *VGI* and *SI*) for quantification of the root postures (Grabov et al., 2005; Figure 2c). To investigate the effects of incubation time and sucrose on these indices, we compared Col-0 root postures under the following treatments: 3-day-old without sucrose (3d−Suc), 3-day-old with sucrose (3d+Suc), 5-day-old without sucrose (5d−Suc) and 5-day-old with sucrose (5d+Suc). The 3d+Suc treatment was identical to the gravitropism assay described in the preceding section. Roots grown under the 5d+Suc treatment were notably longer than those under the 3d+Suc and 5d−Suc treatments (Supplementary Figure S3a). The *VGI* and *SI* of the 5d+Suc roots were lower than those of the roots under the 3d+Suc and 5d−Suc treatments, whereas the *HGI* was invariant among all treatments (Supplementary Figure S3b–d). Under the 3d−Suc, 3d+Suc and 5d−Suc treatments, in which the roots were short, the three indices each showed large variance presumably affected by the direction of germination (Supplementary Figure S3). To mitigate these effects, the following experiments were conducted under the 5d+Suc treatment, which induced longer roots. Under the 5d+Suc treatment, the roots of all genotypes were sufficiently elongated to allow quantitative analysis (Figure 2d). With regard to *HGI*, no differences were observed among the genotypes, including Col-0 (Supplementary Figure S4a; Kruskal–Wallis ANOVA *P*_ANOVA_ = 0.905). In contrast, *SI* and *VGI* were significantly lower for the *xif xik*, *abcb19-101* and *abcb19-102* mutants than for Col-0 (Figure 2e, Supplementary Figure S4b). The *SI* and *VGI* for the triple mutants were similar or slightly lower than those for the *xif xik* and *abcb19* mutants (Figure 2e, Supplementary Figure S4b). These results suggest that *SI* and *VGI* are useful for distinguishing the root posture of the *xif xik* and *abcb19* mutants from that of the wild-type control. However, these root posture indices are determined on the basis of the root length and the coordinates of the root starting and ending points. Therefore, details regarding local bending will be lost if these indices are used.

### 3.3. Roots of the abcb19 mutants had greater CI than roots of the xif xik mutant

**Figure 3.**
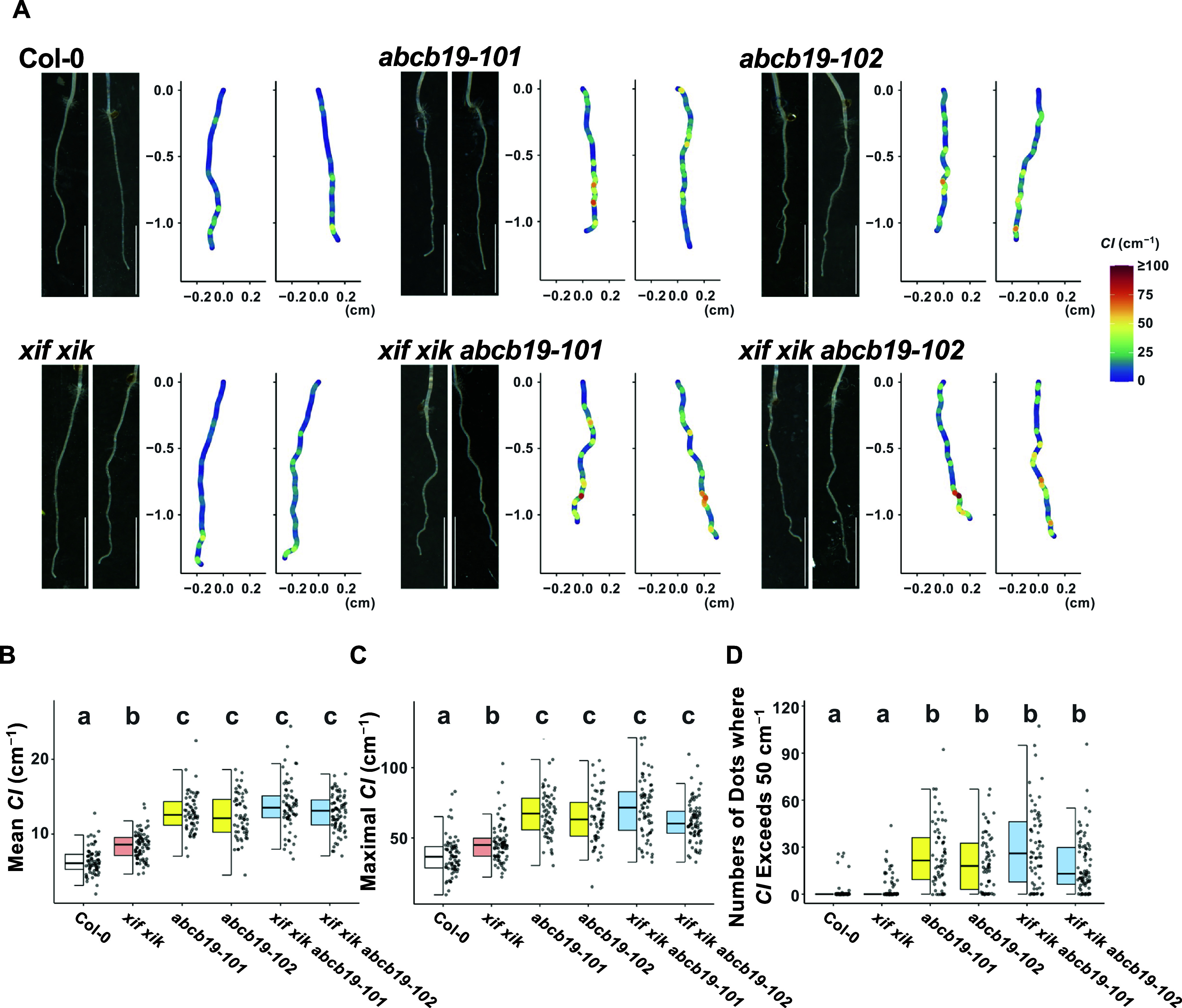
Evaluation of root postures in vertically grown seedlings by the curvature index (*CI*). Wild-type (Col-0), *myosin xif xik* (*xif xik*), *abcb19-101*, *abcb19-102*, *xif xik abcb19-101* and *xif xik abcb19-102* seedlings were grown vertically along the surface of the medium in darkness for 5 days. (a) Images of two representative roots for each genotype and the corresponding coloured diagrams of the *CI*. Scale bars indicate 1 cm and the colour scale is presented on the right. (b–d) Quantitative comparisons of the mean *CI* (b), maximal *CI* (c) and number of dots where *CI* exceeded 50 cm^−1^ (d) are presented as box and dot plots. The bars indicate the sample ranges, each box indicates the first and third quantiles and the bold horizontal line is the median. Sample numbers (*n*) are the same as those in Figure 2d, e. Different letters (a, b and c) indicate a significant difference (*P* < 0.05; Steel–Dwass test following the Kruskal–Wallis ANOVA (*P*_ANOVA_ < 2.2E−16 for all indices)).

The local bending of roots was quantitatively examined by calculating the root *CI*. Representative roots and their *CI* (coloured plots) are presented in Figure 3a. The mean and maximal *CI* were lowest for Col-0 (Figure 3b, c); they were higher for *xif xik* and highest for *abcb19* and the triple mutants (Figure 3b, c). Density histograms of the *CI* for all obtained dots showed that the *CI* distributions of *ABCB19*-deficient mutants were more skewed to higher values than those of Col-0 and the *xif xik* mutant (Supplementary Figure S5). Because Col-0 and *xif xik* had few dots with *CI* > 50 cm^−1^ (Supplementary Figure S5), we set 50 cm^−1^ as the *CI* threshold and counted the number of dots exceeding this threshold per individual root. There were considerably more dots where *CI* exceeded this threshold for all of the *ABCB19*-deficient mutants, whereas almost no dots exceeded this threshold for Col-0 and the *xif xik* mutant (Figure 3d). Notably, unlike *SI*, the mean and maximal *CI* and the number of dots, where *CI* exceeded 50 cm^−1^, were useful for distinguishing between the root postures of the *xif xik* and *abcb19* mutants (Figures 2e and 3b–d).

**Figure 4.**
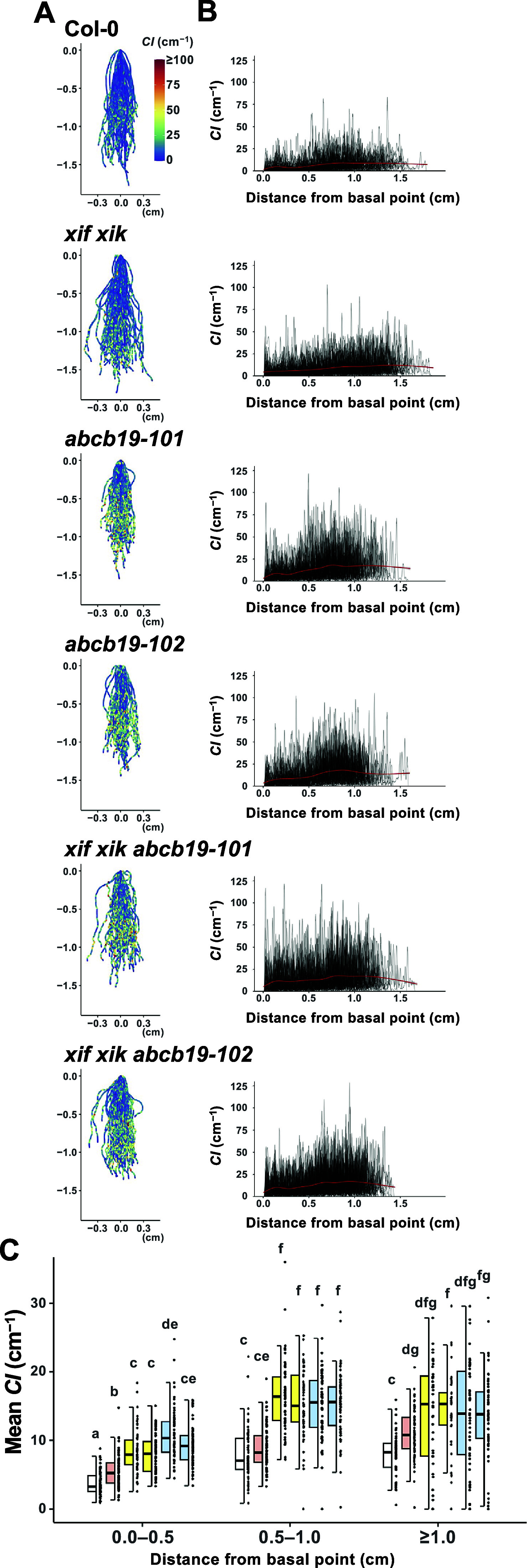
The apical side of the roots showed higher curvature than the basal side. Wild-type (Col-0), *myosin xif xik* (*xif xik*), *abcb19-101*, *abcb19-102*, *xif xik abcb19-101* and *xif xik abcb19-102* seedlings were grown vertically along the surface of the medium in darkness for 5 days. (a) Coloured diagrams of the curvature index (*CI*) of all samples. Sample numbers (*n*) are the same as those in Figure 2d, e. The coordinate (0,0) indicates the basal point (the root–hypocotyl junction) of each root. The colour scale is provided to the right of Col-0. (b) Plots of *CI* versus the distance from the basal point of each root. Red lines represent the generalized additive model regression curves of each genotype. (c) Comparison of the mean *CI* calculated for each of the following distances from the basal point: 0.0–0.5 cm, 0.5–1.0 cm and ≥1.0 cm. The bars indicate the sample ranges, each box indicates the first and third quantiles and the bold horizontal line is the median. Different lowercase letters indicate a significant difference (*P* < 0.05; Steel–Dwass test following the Kruskal–Wallis ANOVA (*P*_ANOVA_ < 2.2E−16)).

### 3.4. The apical side of the roots had a higher CI than the basal side

We observed that *CI* tended to be relatively low and high on the basal and apical sides of the roots, respectively, for all of the analysed genotypes. The root postures of all samples are presented in Figure 4a, with colours indicating *CI*. In addition, “distance from the basal point (the root–hypocotyl junction)” versus *CI* was plotted (Figure 4b). For simplicity, the roots were divided into three compartments (0.0–0.5 cm, 0.5–1.0 cm and ≥1.0 cm from the basal point) and then the mean *CI* was calculated. In each compartment, the mean *CI* was lower for *xif xik* than for *abcb19* and lowest for Col-0 (Figure 4c). Although the mean *CI* was higher for the triple mutants than for *abcb19* in the 0.0–0.5 cm compartment, there was almost no difference in the mean *CI* between the triple mutants and *abcb19* in the compartments ≥0.5 cm from the basal point (Figure 4c). Similar results were observed in the mean *CI* for the whole root length (Figure 3b). Comparison of the compartments revealed that the mean *CI* was lowest in the 0.0–0.5 cm compartment for all genotypes, whereas the mean *CI* in the 0.5–1.0 cm and ≥1.0 cm compartments was almost the same for the analysed genotypes, with the exception of *xif xik*, in which the mean *CI* was highest in the ≥1.0 cm compartment (Figure 4c). Moreover, the *CI* in the 0.5–1.0 cm compartment of the *ABCB19*-deficient mutants was higher than the mean *CI* in the ≥1.0 cm compartment of the *xif xik* mutant.

## 4. Discussion

In the current study, we used four indices to evaluate root posture, of which *HGI*, *VGI* and *SI* are determined only on the basis of the root length and the coordinates of the root starting and ending points (Grabov et al., 2005). When the plants were grown vertically, the *xif xik abcb19* triple mutants had similar or slightly lower *SI* than the *xif xik* and *abcb19* mutants (Figure 2e), indicative of the utility of *SI* for differentiating the root posture of *xif xik abcb19* from that of *xif xik* and *abcb19*. Because of their simplicity, these indices are useful for processing large amounts of root data. However, these three indices were unable to distinguish between the *xif xik* and *abcb19* roots with apparently similar phenotypes (Figure 2e and Supplementary Figure S3). We attempted to quantify the fine root bending using *CI*, which reflects the whole root posture. The *xif xik* roots had a higher *CI* than the Col-0 roots, but a lower *CI* than the *abcb19* roots (Figures 3 and 4). These findings indicate that combination of *SI* and *CI* may enable the classification of a variety of root postures. Because verification of the straightening ability is labour-intensive and time-consuming (e.g., involving a clinostat analysis) (Okamoto et al., 2015; Ueda & Hara-Nishimura, 2019), screening for straightening mutants is extremely difficult. The present method, which evaluates root posture at a single time point, is applicable for screening with less effort. Certain automatic systems enable measurement of root gravitropic angle or root traits (Clark et al., 2013; Serre & Fendrych, 2022). In future, we intend to develop an automatic system for the present method. In the current system, the plates are exposed to light and the gravitational direction is changed to enable scanning (see Materials and Methods), which could alter the root posture. The next step will be to overcome these issues, for example, by using infrared cameras, which would allow measurements in a time series that could lead to a more detailed evaluation.

The present study provides insights into control of plant posture using the *xif xik* and *abcb19* mutants. Plant posture is controlled by environmental and genetic factors. During the gravitropic response, plant posture is believed to be established by many processes, including gravity sensing, auxin redistribution leading to differential growth, organ bending, posture sensing and organ straightening. Aberrantly enhanced organ bending in response to gravity is due to an abnormality in one or more of these processes. In the present study, the *xif xik* and *abcb19* roots with similar enhanced gravitropic bending differed in *CI* (Figures 1, 3 and 4), and the *xif xik abcb19* triple mutants showed greater gravitropic bending than the *xif xik* and *abcb19* mutants (Figure 1), suggesting that MYOSIN XIs and ABCB19 may affect different gravitropism-related processes. The *SI* was similar for the *xif xik* and *abcb19* mutants (Figure 2), whereas the *CI* for *abcb19* was higher than for *xif xik* (Figures 3 and 4). These results imply that the *xif xik* roots had large and gradual curves, whereas the *abcb19* roots had small and sharp curves. Furthermore, the *SI* for the triple mutants was similar or slightly lower than for *xif xik* and *abcb19*, but the *CI* for the triple mutants was similar to that for *abcb19* (Figures 2, 3 and 4). This may be because the roots of triple mutants have large and sharp curves. Previous research indicates that ABCB19 is essential for maintaining the auxin maxima at the root tip; when ABCB19 is deficient, the auxin accumulation at the root tip extends basally (upwards) (Lewis et al., 2007). Additionally, if the increased auxin contents in *abcb19* roots are not distributed symmetrically, the resulting imbalanced cell elongation rates will lead to wavy roots (Lewis et al., 2007). MYOSIN XIf and MYOSIN XIk are crucial for straightening organs (Okamoto et al., 2015; Tsugawa et al., 2023), possibly through posture sensing or posture restoration, although how MYOSIN XI proteins function in these processes is unknown. At this point, we cannot rule out the possibility that ABCB19 also contributes to organ straightening. In the present study, the *CI* for the *xif xik* roots clearly increased when ABCB19 was deficient (Figures 3 and 4). However, the *CI* for the *abcb19* roots was unaffected by deficiencies in both MYOSIN XIf and MYOSIN XIk (Figures 3 and 4). These results suggest that the deficiency in ABCB19 had a greater effect than the simultaneous deficiency in MYOSIN XIf and MYOSIN XIk on the *CI*.

## Supporting information

Yagi et al. supplementary material Yagi et al. supplementary material

## Data Availability

The data including raw images of plants and *Arabidopsis thaliana* seeds reported in this study are available from the corresponding author upon request.

## Supplementary material

The supplementary material for this article can be found at http://doi.org/10.1017/qpb.2024.12.
