# Supplementary material for: Quantitative analysis of the root posture of Arabidopsis thaliana mutants with wavy roots
Source: Quant Plant Biol. 2024 Nov 25;5:e9. doi: 10.1017/qpb.2024.12 (PMC11706685; doi:10.1017/qpb.2024.12)
Supplement: Yagi et al. supplementary material [file S2632882824000122sup001.pdf]

Supplementary Fig. S1

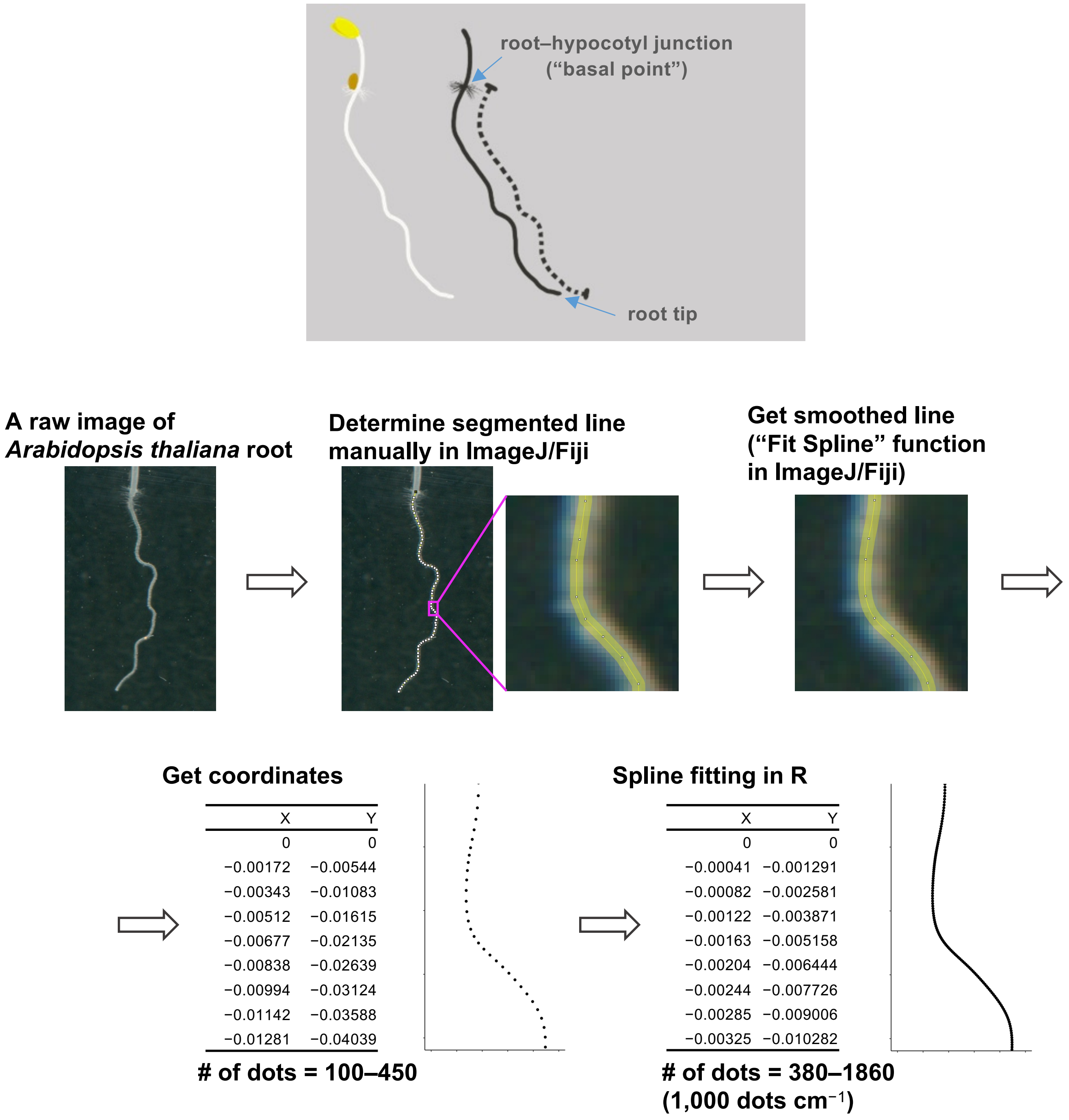

Supplementary Fig. S1. Procedure to obtain root coordinates in this study. Five-day-old etiolated *Arabidopsis thaliana* seedlings grown almost vertically (pitches were 80–90°) on medium in plates in darkness were placed under light and photographed. The “segmented line” along the roots (from root–hypocotyl junction to root tip as depicted above) was determined manually using ImageJ/Fiji software and transformed into “smoothed line” using the Fiji function “Fit Spline.” In total, 100–450 dots were marked along the root and then the coordinate data were exported to R. Finally, the sequential coordinates were fitted via spline interpolation to comprise 1,000 dots cm^{-1} using the “smoothr” R package.

Supplementary Fig. S2

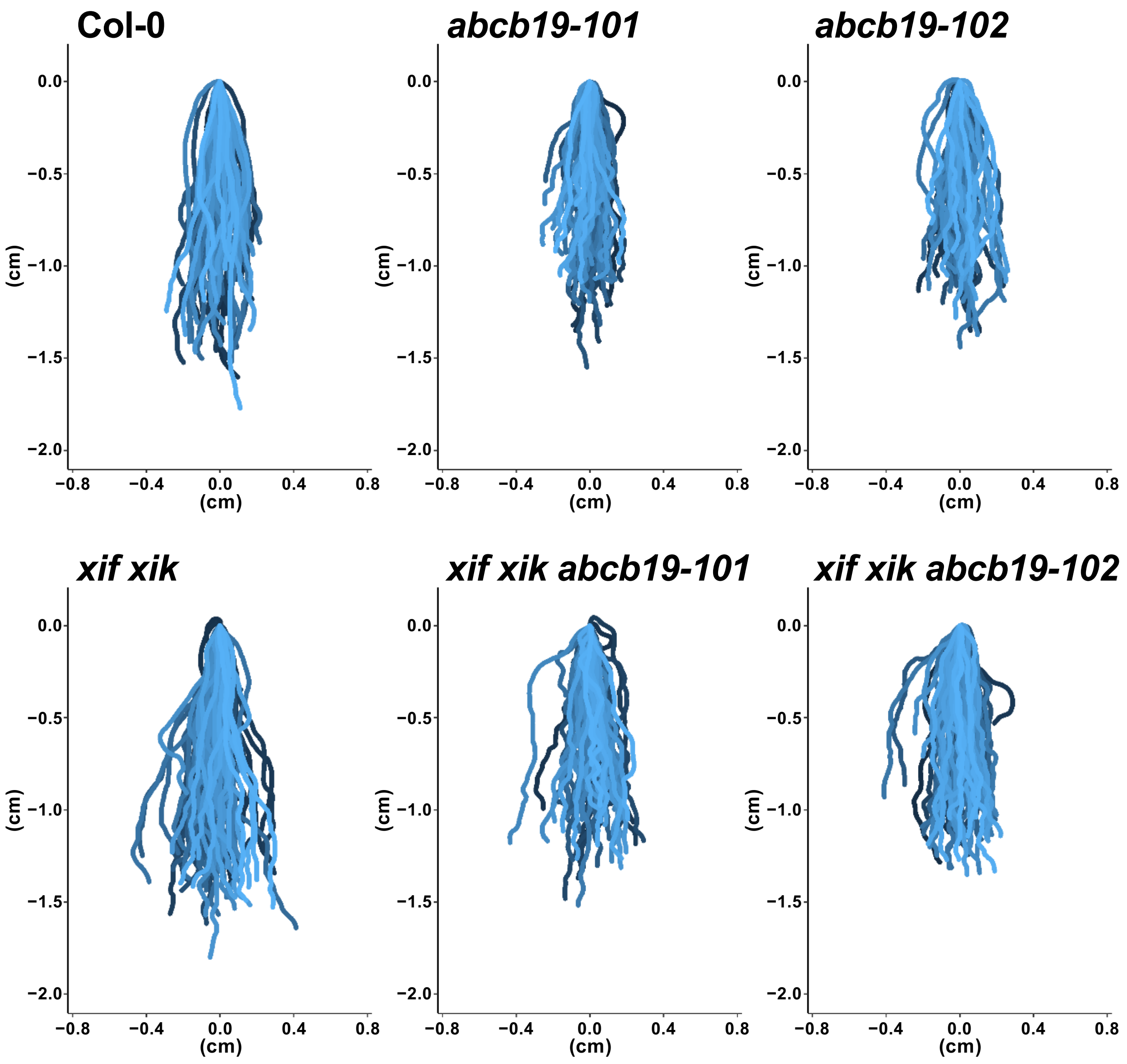

Supplementary Fig. S2. Posture plots of all individual roots for each genotype. Wild-type (Col-0), *myosin xif xik* (*xif xik*), *abcb19-101*, *abcb19-102*, *xif xik abcb19-101*, and *xif xik abcb19-102* seedlings were grown vertically along the surface of the medium in darkness for 5 days. The coordinate (0,0) indicates the basal point (the root–hypocotyl junction) of each root. The number of samples (*n*) are as follows: 71 (Col-0), 77 (*xif xik*), 62 (*abcb19-101*), 59 (*abcb19-102*), 66 (*xif xik abcb19-101*), and 78 (*xif xik abcb19-102*).

Supplementary Fig. S3

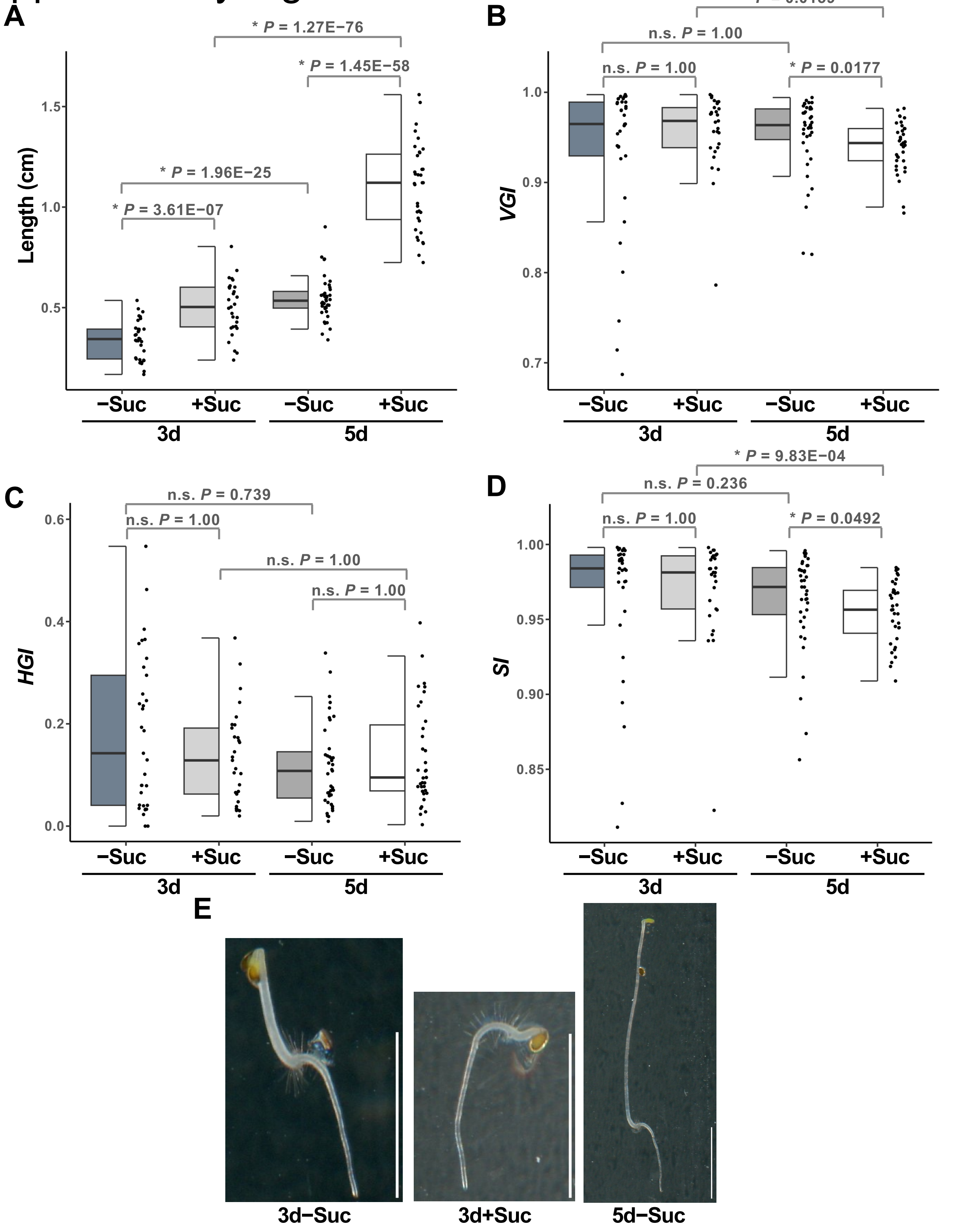

Supplementary Fig. S3. Effects of incubation time and sucrose on the root postures. Wild-type (Col-0) seedlings were grown vertically along the surface of the medium supplemented with or without sucrose in darkness for 3 or 5 days. Length (**A**), *VGI* (**B**), *HGI* (**C**), and *SI* (**D**) are presented as box and dot plots. The bars indicate the sample ranges, each box indicates the 1st and 3rd quantiles, and the bold horizontal line is the median. The number of samples (*n*) are as follows: 33 (3d−Suc), 29 (3d+Suc), 39 (5d−Suc), and 35 (5d+Suc). Statistical analysis was performed with the Brunner–Munzel test with the Bonferroni correction (*, $P < 0.05$; n.s., not significant). (**E**) Representative images of horizontally germinated plants. Scale bars indicate 0.5 cm.

Supplementary Fig. S4

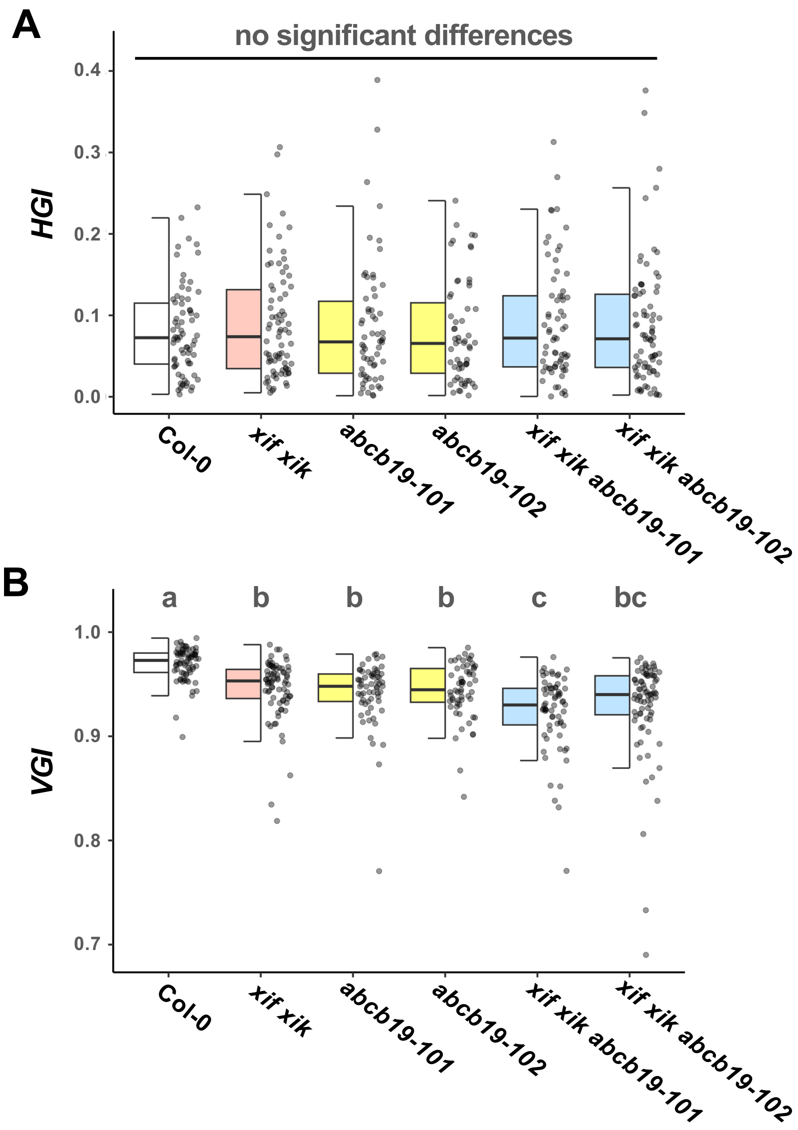

Supplementary Fig. S4. Quantitative comparisons of horizontal growth index (*HGI*) and vertical growth index (*VGI*).
Wild-type (Col-0), *myosin xif xik* (*xif xik*), *abcb19-101*, *abcb19-102*, *xif xik abcb19-101*, and *xif xik abcb19-102* seedlings were grown vertically along the surface of the medium in darkness for 5 days. *HGI* (**A**) and *VGI* (**B**) are presented as box and dot plots. The bars indicate the sample ranges, each box indicates the 1st and 3rd quantiles, and the bold horizontal line is the median. The number of samples (*n*) are as follows: 71 (Col-0), 77 (*xif xik*), 62 (*abcb19-101*), 59 (*abcb19-102*), 66 (*xif xik abcb19-101*), and 78 (*xif xik abcb19-102*). There are no differences among the genotypes in *HGI* (**A**) (Kruskal–Wallis ANOVA test $P_{ANOVA} = 0.905$). Different lowercase letters in *VGI* (**B**) indicate a significant difference [$P < 0.05$. Steel–Dwass test following the Kruskal–Wallis ANOVA ($P_{ANOVA} < 2.2E{-}16$)].

Supplementary Fig. S5

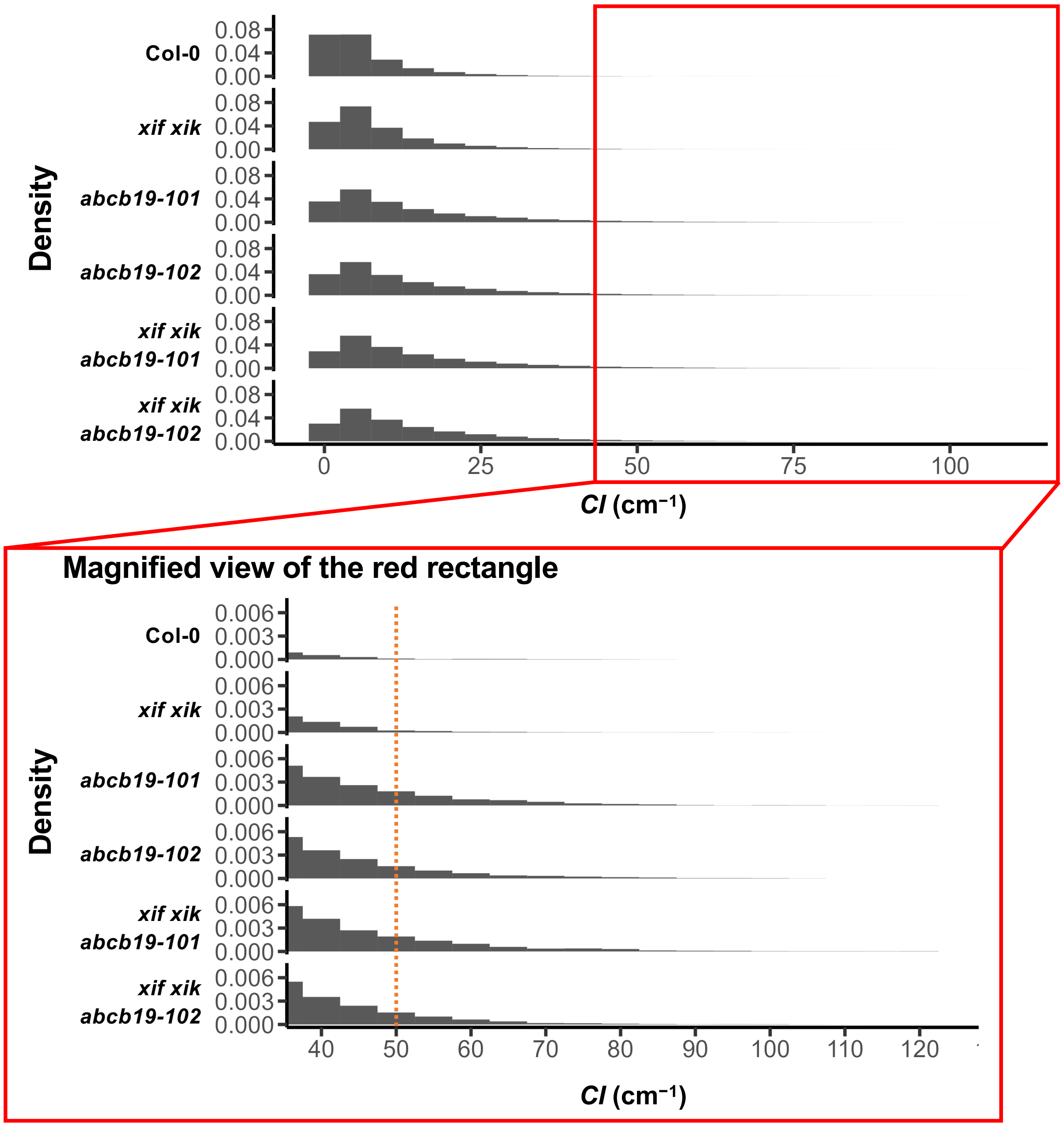

Supplementary Fig. S5. Density histogram of curvature index (*CI*).
The *CI* from all obtained dots plotted as density histograms. A magnified view of the red rectangle in the upper plot is shown below.
