# Supplementary material for: Quantitative analysis of the root posture of Arabidopsis thaliana mutants with wavy roots
Source: Quant Plant Biol. 2024 Nov 25;5:e9. doi: 10.1017/qpb.2024.12 (PMC11706685; doi:10.1017/qpb.2024.12)
Supplement: Peer review history

# Editor report: Recommendation: Quantitative analysis of the root posture of *Arabidopsis thaliana* mutants with wavy roots — R0/PR2

**DOI:** 10.1017/qpb.2024.12.pr2

**Affiliations:** Researcg group in Biophysics and Morphogenesis of Plants, University of Silesia in Katowice, Poland

The Reviewers appreciate the significance of the presented results and methods. They also give a number of valuable suggestions and comments that need to be addressed by the Authors. My recommendation is therefore a major revision.

# Editor report: Decision: Quantitative analysis of the root posture of *Arabidopsis thaliana* mutants with wavy roots — R0/PR3

**DOI:** 10.1017/qpb.2024.12.pr3

**Affiliations:** RDP, ENS de Lyon, France

No accompanying comment.

# Editor report: Recommendation: Quantitative analysis of the root posture of *Arabidopsis thaliana* mutants with wavy roots — R1/PR5

**DOI:** 10.1017/qpb.2024.12.pr5

**Affiliations:** Researcg group in Biophysics and Morphogenesis of Plants, University of Silesia in Katowice, Poland

The manuscript has been improved but there remains a problem with statistics methods that were used by the Authors. They are explained in detail by the Reviewer #2 who is an expert in statistics, who also provided specific advice on which tests the Authors can use. My recommendation is therefore minor revision. I strongly advice that the statistics is corrected in the final manuscript version.

# Editor report: Decision: Quantitative analysis of the root posture of *Arabidopsis thaliana* mutants with wavy roots — R1/PR6

**DOI:** 10.1017/qpb.2024.12.pr6

**Affiliations:** RDP, ENS de Lyon, France

No accompanying comment.

# Editor report: Recommendation: Quantitative analysis of the root posture of *Arabidopsis thaliana* mutants with wavy roots — R2/PR8

**DOI:** 10.1017/qpb.2024.12.pr8

**Affiliations:** Researcg group in Biophysics and Morphogenesis of Plants, University of Silesia in Katowice, Poland

The statistics used in the manuscript has been modified according to the Reviewer’s suggestions, and the manuscript is now ready for publication.

# Editor report: Decision: Quantitative analysis of the root posture of *Arabidopsis thaliana* mutants with wavy roots — R2/PR9

**DOI:** 10.1017/qpb.2024.12.pr9

**Affiliations:** RDP, ENS de Lyon, France

No accompanying comment.
